# Health status in the ambulance services: a systematic review

**DOI:** 10.1186/1472-6963-6-82

**Published:** 2006-07-03

**Authors:** Tom Sterud, Øivind Ekeberg, Erlend Hem

**Affiliations:** 1 Department of Behavioural Sciences in Medicine, Institute of Basic Medical Sciences, Faculty of Medicine, University of Oslo, PO Box 1111 Blindern, NO-0317 Oslo, Norway

## Abstract

**Background:**

Researchers have become increasingly aware that ambulance personnel may be at risk of developing work-related health problems. This article systematically explores the literature on health problems and work-related and individual health predictors in the ambulance services.

**Methods:**

We identified the relevant empirical literature by searching several electronic databases including Medline, EMBASE, PsychINFO, CINAHL, and ISI Web of Science. Other relevant sources were identified through reference lists and other relevant studies known by the research group.

**Results:**

Forty-nine studies are included in this review. Our analysis shows that ambulance workers have a higher standardized mortality rate, higher level of fatal accidents, higher level of accident injuries and a higher standardized early retirement on medical grounds than the general working population and workers in other health occupations. Ambulance workers also seem to have more musculoskeletal problems than the general population. These conclusions are preliminary at present because each is based on a single study. More studies have addressed mental health problems. The prevalence of post-traumatic stress symptom caseness was > 20% in five of seven studies, and similarly high prevalence rates were reported for anxiety and general psychopathology in four of five studies. However, it is unclear whether ambulance personnel suffer from more mental health problems than the general working population.

**Conclusion:**

Several indicators suggest that workers in the ambulance services experience more health problems than the general working population and workers in other health occupations. Several methodological challenges, such as small sample sizes, non-representative samples, and lack of comparisons with normative data limit the interpretation of many studies. More coordinated research and replication are needed to compare data across studies. We discuss some strategies for future research.

## Background

Until recently, occupational health within the ambulance services has received relatively little attention from researchers. In the past few years, researchers have become increasingly aware that ambulance personnel may be at risk of developing work-related health problems [1,2]. This is the first systematic review to address the health status of workers in the ambulance services.

Research on health in the ambulance services has been based on the assumption that such work is inherently stressful [3]. Ambulance workers frequently have to take rapid action and provide medical care under life-and-death circumstances in unfamiliar and inconvenient circumstances while being scrutinized by bystanders and relatives [4]. Moreover, they often have to deal with unpredictable and non-specific threats, such as the possibility of contracting diseases from patients or being attacked by mentally unstable or violent patients [5]. Ambulance personnel also must attend to non-emergency work, such as transporting and providing appropriate care to chronically and terminally ill patients, which imposes different emotional demands and which might be experienced as more emotionally exhausting than more sensational events [6]. A review addressing the relation between stress and strain in ambulance workers published 10 years ago [7] concluded that more research is needed to evaluate the prevalence of post traumatic stress disorder among ambulance personnel, and to compare their level of depression and anxiety to the general population [7].

A number of studies have been published with a much broader health scope than the stress related health problems addressed previously [7]. Hence, an updated review is needed to evaluate ambulance workers' health status.

Our objective was to present an updated review addressing health problems among ambulance personnel. The specific research questions are:

1. Do ambulance personnel have more health problems compared with other occupational groups?

2. What is the evidence for negative health consequences within the ambulance services as a result of stressful/harmful work conditions and individual differences (e.g., personality and coping behaviour)?

## Methods

### Search strategy

The relevant empirical literature was identified by searching several electronic databases: Medline (Ovid) (1966 to 2005, week 48), EMBASE (1980 to 2005, week 48), PsychINFO (1972 to 2005, week 48), CINAHL (1982 to 2005, week 48), and ISI Web of Science (1980 to 2005, week 48). The search was performed by cross-referencing the words "ambulance personnel", "ambulance worker", "ambulance-men", "paramedics", "emergency medical technicians" or "ems personnel" with "mental health", "stress", "stress disorders, post-traumatic", "stress, psychological", "depression", "anxiety", "burnout", "physical health", "health status", "sick leave", "mortality", "blood pressure", "heart rate", "retirement" or "wounds and injuries", "cancer", "lung diseases", "gastrointestinal diseases", "accidents, occupational", "occupational disease", "cardiovascular diseases", "musculoskeletal diseases", "(substance abuse detection or substance related disorders)", "communicable diseases", or "respiration disorders".

Other relevant sources were identified through reference lists and other relevant studies known by the research group [8-11].

### Inclusion and exclusion criteria

All the identified abstracts were reviewed. Then the full text versions of all potentially relevant papers were reviewed and formally assessed for inclusion by the main author. Reasons for the exclusion of papers are reported in the results section. Languages were not limited. The inclusion criteria were:

1. An original study published in a peer-reviewed journal, where an abstract was reported in the databases, in which either health status separately or the relation between health and stressful working conditions or individual differences in the ambulance services was assessed.

2. The study was published after 1966, as an arbitrary limit.

3. Among the studies addressing the relation between stress and strain in the ambulance service only studies not included in the previous review [7] were chosen for further examination.

### Data abstraction and quality assessment

Once a study was included, the following data were extracted by one of the authors (TS): authors; year of publication; site; aims of the study; study design; key explanatory variables, outcome variables; statistical methods; and main results. The information was summed up in a table [see Additional file 1]. Studies were ranged according to level of evidence by the authors based on methodological considerations, such as sample bias, lack of comparison groups, and design. For the first research question, studies that compared ambulance workers with other groups of workers using the same methods and instruments were considered most adequate, and for the second research question, prospective studies were considered most adequate. The highest quality studies are described first in the text under each headline.

## Results

The search resulted in 573 hits in the Ovid Medline database, of which 39 fulfilled the inclusion criteria [1,3,12-48].

Most studies excluded were "short reports" where abstracts were not given (n = 245), others had a patient-centred approach (n = 193) or focused on stress management or debriefing of ambulance personnel (n = 11). Other studies were excluded because of no relevant health outcome (n = 28), no specified ambulance sample (n = 10), a focus on leisure activities (n = 2), or not reporting original research (n = 5). Studies dealing with the aftermath of the "9/11" attack (n = 4) were excluded because of the special circumstances and the inclusion of volunteers. Several studies had a focus on infectious diseases, mainly hepatitis (n = 19). These studies have been addressed in two reviews [49,50], which will be briefly presented here. Two studies were excluded [51,52] because the same findings were reported in a follow-up study [53]. Fifteen of the studies were excluded because they had been reported in the previous review [7].

Only five relevant studies were identified in other databases [2,54-57], four were known by the research group [8-11], and one other study [53] was identified in the reference lists. Thus, 49 studies were included in this review.

The studies were from the USA (21), the United Kingdom (6)/Northern Ireland (3), Sweden (4), the Netherlands (3), Canada (3), Australia (2), Wales (2), France (1) Germany (1), New Zealand (1), Japan (1) and Scotland (1). The previous review included only 23 original studies [7], whereof 15 were identified in our Medline search, and two in other databases. Although available in the searched databases, six of the studies were not identified by our search strategy because four studies did not have a specified ambulance sample [58-61], one study did not give an abstract [62], and one study was not indexed in the databases [63]. We identified four relevant studies addressing the relations between stress and strain [18,30,35,42] published before 1995 that were not included in the previous review [7]. The search strategy was not reported in the previous review. However, because the identified studies overlap considerably with our review, the conclusions about the correlations between stress and strain symptoms seem to be based on most of the relevant studies published before 1995. We discuss their main findings in light of more recent research.

### Post-traumatic stress disorder symptoms

Several studies have addressed the prevalence of post-traumatic stress disorder (PTSD) symptoms in the ambulance services. A study from a region in Sweden (n = 362 [sample size]/72.4% [response rate]) is the largest study, and claimed to be representative [29,56]. The prevalence of PTSD symptoms among ambulance workers was 12% on the Post-traumatic Symptom Scale (PTSS-10), and 15% or 21.5% using different criteria on the Impact of Event Scale (IES). The authors concluded that the prevalence of PTSD symptoms among ambulance personnel (21.5%) was much higher than in the general population in Sweden (2.6%). A study from an urban area in the USA (n = 225) used the Minnesota Multiphasic Personality Inventory-2 PTSD Scale (MMPI-2 PK) and reported a 20% prevalence of PTSD symptoms among ambulance workers, which was higher than the prevalence of 5% for men and 10% for women in the general population [26]. However, none of these studies reported confidence intervals (CI) or *t *values, making the comparisons questionable. Similar high prevalence rates measured by the IES were reported in studies from Canada [37] and Scotland [14], and in a study from the UK that used the Post-traumatic Diagnostic Scale [1]. A slightly lower prevalence was reported in another study from Canada (15.9%) [2], and in a study from the Netherlands (12%) [45].

In conclusion, the prevalence of PTSD symptoms is consistently high in the regular ambulance services, about 20% of ambulance workers in five of seven studies. However, no study has adequately tested whether ambulance workers have a higher risk than those in other occupational groups.

#### Acute stressors and coping

Two of the studies focusing on acute stressors had a longitudinal design, both of which were conducted in the USA. The PTSD symptom responses of rescue workers (n = 322, including paramedics, firefighters, and police) were measured using self-administered questionnaires 1.5 years (T1) and 3.5 years (T2) after critical incidents[53]. Despite modest improvements in symptoms at follow-up, rescue workers were at risk for chronic symptomatic distress after exposure to critical incidents. The degree of exposure, peri-traumatic dissociation, fewer years of experience, external locus of control, and poor social support predicted higher levels of symptoms at T2 when the level of distress at T1 was controlled. In another study on firefighters and paramedics[16], avoidance coping was significantly associated with an increase in PTSD symptoms during a six-month prospective follow-up period. The number of duty-related traumatic incidents did not have a significant effect.

Three cross-sectional studies have reported associations between coping, i.e. suppression of emotions at a neurotic level [46], wishful thinking [23], low sense of coherence [56], ego defence mechanisms (displacement, regression, and projection) [26], and more PTSD symptoms.

Other studies have focused on the psychosocial work environment reporting significant correlations between frequency of incident stressors and degree of organizational stress [17], degree of operational hassles [54], degree of emotional demands and poor communication [45], length of review following a critical event resulting in loss of life [2], and PTSD symptoms.

In conclusion, four studies have identified emotional ways of coping [16,23,46,53,56] significantly related to more PTSD symptoms, but no studies have been able to identify any protective coping strategies. Degree of work-related stress was related to more PTSD symptoms in three studies [17,45,54]. However, it is not yet possible to conclude on the relationship between psychosocial work factors and individual coping in explaining PTSD symptoms, and the specific work-related stressors and coping dimensions that reliably predict adverse stress reactions among ambulance workers are still uncertain.

### Burnout

A study from the Netherlands [45] used the Maslach Burnout Inventory to investigate the prevalence of burnout in workers from 10 regions (n = 221/56%) and found a higher risk for burnout in ambulance workers (8.6%) than in the general working population (5.3%). However, the CI and significance level were not reported. The percentages of workers with high scores on the separate dimensions were reported as 12% for emotional exhaustion (EE), 18% for depersonalization (DP), and 16% for low personal accomplishment (PA). The study reported a significantly higher prevalence of fatigue (10%), as measured by the Checklist Individual Strength, in ambulance workers, but the prevalence in the reference group was not reported. A study from a single service in the USA (n = 69) reported an opposite result and concluded that the average burnout scores in ambulance workers were slightly but not significantly lower than the national average [47]. However, this conclusion was based on a small and non-representative sample. A study from a Scottish regional ambulance service (n = 160/69%) reported the percentages of workers with high scores on the Maslach Burnout Inventory for the separate scales as 26% for EE, 36% for low PA, and 22% for DP, but did not report confidence intervals [14].

Hence, no clear conclusion can be made about the prevalence of burnout in the ambulance services, and it is an open question whether burnout levels are higher than in the general working population.

### Depression and anxiety

The level of anxiety and depression, as measured by Spielberger's State-Trait Anxiety Inventory, was lower in ambulance workers from a county-wide service in the USA (n = 63/81%) than in a normative sample of working males [34]. However, because these comparisons were based on a small and non-representative sample and the variance scores and prevalence were not reported, the mean scores might have obscured important individual differences between the comparison groups.

In a relatively large study from a single organization in Wales (n = 617/60%) [1], the prevalence of anxiety caseness was 22% and depression caseness was 10%, as measured with the Hospital Anxiety and Depression Scale (cut-off > 11). Three other studies reported a similarly high prevalence of psychological distress (> 20%), as measured by the General Health Questionnaire [GHQ-12] [14,23,43]. A lower prevalence (2.1%) of severe symptoms, as measured with Beck's Depression Inventory, was reported in a study from a single ambulance service in Canada [37].

In conclusion, four out of five studies claimed that as many as 20% of workers in the ambulance services suffer from psychopathological problems, but it is unknown whether this prevalence differs from that in the working populations in the respective countries.

#### Work-related and individual correlates

Both chronic and acute stressors were addressed in a one-year follow-up study of ambulance personnel from 10 regions in Holland (n = 142/36% at T2) comprising a representative sample of the rural-urban distribution [45]. Lack of support from co-workers and supervisors at T1 predicted more burnout symptoms and general fatigue at T2. These findings were significant after controlling for symptoms at T1. Self-reported acute stressors were associated with fatigue, and burnout at T1, but did not predict health symptoms at T2. Lack of autonomy and financial reward were not significant predictors. In a prospective study from the USA (n = 65/33% at T2), high work group and supervisor support predicted less work-related stress at the six-month follow-up (T2), and an increase in stress from T1 to T2 predicted an increase in psychological distress [39].

Two nationwide cross-sectional studies were identified. In a recent study from Sweden (n = 1189/79%) [12], worry about job conditions and psychological demands for both genders, and low social support for men only, were significantly associated with more sleeping problems, headache- and stomach symptoms. Unfortunately, the health data was not based on standardized instruments. A study from New Zealand (n = 686/46%, 232 ambulance workers) [54] showed that stress levels did not differ significantly between police, firemen, and ambulance workers. Operational stressors were associated with more trauma symptomatology and psychological strain in all groups. In contrast, organizational hassles were not significantly correlated with psychological strain in ambulance personnel.

In accordance with these results, three cross-sectional studies have reported significant relations between high job stress and psychological strain [15,17,19]. Four studies have reported low social support to be associated with more mental health problems [3,15,17,57]. However, in a study from Canada, social support was not significantly associated with mental health problems when individual factors were included in the multiple regression models [2].

A few studies have focused on individual factors and two prospective studies were identified. In a follow-up study (n = 70) in the ambulance service in North England, external control and type A personality predicted more mental health symptoms at the six-month follow-up, but work-related stressors had no significant effect on symptoms [48]. A one-month follow-up study from the USA reported that coping was more highly correlated with psychological strain at T2 than with frequency of events, but the data were not presented [19]. The highest significant correlations were between accepting responsibility (self-criticism and self-blame), escape-avoidance coping, confrontive coping (i.e., handling stressors with aggression and hostility), and psychological strain; correlation coefficients ranged from 0.3 to 0.4.

In conclusion, work-related stress was associated with mental health problems in seven studies [12,15,17,19,39,45,54]. However, two studies reported that personality factors [48] and coping [20] were more highly correlated with psychological strain than work- related stress were. Low support was associated with mental health problems in six studies [3,15,17,39,45,57]. In one study, separate analysis for men and women showed a significant association only for men [12], and one study found no significant effect of social support when coping was adjusted for [48].

### Medical impairment and early retirement

A regional study between 1988 and 1992 in Northern Ireland reported a high standardized rate of early retirement for medical reasons, after controlling for age and sex, among ambulance workers (636, 95%CI: 558–714) compared with the group with the second highest ratio, manual workers (164, 95%CI: 149–179), non-manual workers (38, 95%CI: 25–52), and nursing workers (91, 95%CI: 75–107) [40]. The main causes of retirement were musculoskeletal, circulatory, and mental disorders, although the main causes did not differ significantly between occupational groups. However, ambulance personnel were more likely to retire because of circulatory and mental problems, especially alcohol abuse, than manual workers [41].

A register-based study from Sweden found that paramedics had a higher rate of permanent medical impairment (2.2 per 1000 gainfully employed people per year) than other health service workers (mean 0.38 per 1000), but the incidence measured in paramedics was based on a small sample size of five [9].

Hence, ambulance workers seem to be at a relatively higher risk of permanent medical impairment and early retirement on medical grounds than other occupational groups.

### Somatic health

A large study from Japan (n = 1551/76.9%) addressed self-reported physical health problems. Two-thirds of the emergency medical technicians reported lower back problems, compared with one-quarter of the general working population. Nearly one-third of workers in the ambulance services also reported problems with the neck, shoulders, and knees [36]. In a study from the UK, ambulance workers (n = 52) reported more physical health problems on average (15.1 v. 13.8) than the general working population[55]. Unfortunately, none of the studies reported confidence intervals (CI) or *t *values, making the comparisons questionable.

A small study from the USA, based on a convenience sample, found that 48% reported back pain within the last 6 months, but not all (39%) was related to emergency- work [22]. In a recent nationwide representative study from Sweden (N = 1189/79%), one-fourth of the female and one-fifth of the male ambulance personnel reported two ore more health complaints (headache, stomach symptoms, and sleeping problems) sometimes or often. However, no comparative data was reported [12].

In a study from an ambulance service in Northern Ireland [18], ambulance workers had significantly higher average systolic and diastolic blood pressure (n = 105/46%) than the general population. Twenty-one percent of the ambulance workers had systolic blood pressure > 140 mm Hg and seven percent had systolic blood pressure > 160 mm Hg, the high-risk threshold; however, caseness was not presented for the general population.

Three studies described the associations between work-related stressors and physiological stress symptoms. Ambulance workers exhibited higher physiological arousal (e.g., elevated heart rate and blood pressure) [42], salivary cortisol response [10], and noradrenalin and adrenalin levels [30] when running calls and during more severe emergency incidents than during less severe incidents.

A review from 2001 on the risk of acquiring hepatitis B or C among public safety workers included 72 studies. Fulltime EMS providers were reported to have rates of parenteral (needlestick) and nonparenteral exposure to blood and body fluids similar to those of other health care workers, including doctors and nurses. The authors concluded that EMS personnel, including firefighters, may be at risk of contracting hepatitis B as a result of their work exposure, although the risk was low. Data did not support increased prevalence of hepatitis C. Most studies were based on a convenience sample, and therefore the data might not be representative [49]. The same conclusion was made in a recent unsystematic review without reporting the number of included studies [50].

In conclusion, three studies have reported that ambulance personnel have more somatic health problems, i.e., higher blood pressure [18] and more self-reported musculoskeletal [36] and physical health [55] problems than the general population. There is no clear evidence for a heightened risk of hepatitis in ambulance personnel.

### Mortality, fatal accidents and injuries

The largest study on mortality among ambulance workers is from the UK and covers the period 1979–83 [8]. Ambulance workers (n = 714) had a slightly higher standardized mortality rate (SMR; 109, 95%CI: 101–117) compared with the national average (100). However, the SMR in ambulance workers was slightly lower than that of the general population within the same social class (121, 95%CI: 21–122). Ambulance workers had a significantly higher risk of dying from ischemic heart disease, the most frequent cause of death among ambulance personnel, and all cancers than the national average. Another study based on national databases in the USA over the period 1992–97 found a relatively higher number of fatal accidents among ambulance workers (12.7 per 100,000) than in the general working population (5 per 100,000), and at levels similar to those in police (14.2 per 100,000) and firefighters (16.2 per 100,000) [31]. Most accidents were ground transportation related and air ambulance crash fatalities. There were, however, some uncertainties about both the coding of injuries and the estimates of the total number of ambulance workers.

A study from the USA of occupational injuries among fulltime ambulance personnel in two urban areas, reported a total injury-rate of 34.6 per 100 fulltime workers. The relative risk for injury was slightly higher compared to firefighters (RR = 1.5, 95%CI: 1.35–1.72), and higher than the average in the health services (RR = 5.8, 95%CI: 5.12–6.49), and the national average (RR = 7.0, 95%CI: 6.22–7.87). Sprains and strains was the leading case type, and the back was most often injured [64]. In two other studies from urban US settings, an injury rate of 50 cases per 100 fulltime workers for men and 86 cases per 100 fulltime workers for women [28], and an injury rate of 115 per 100 fulltime worker were reported [25]. Musculoskeletal injuries and low back pain were the most commonly reported injuries.

A study from Philadelphia, USA reported that 4% of all injuries were caused by assaults (2.3% from intentional assaults), of which 32% resulted in time lost from work [33]. A study from Paris found that one or more assaults were recounted by 23% of the ambulance personnel during their career, 4% resulted in sick leave and 4% resulted in PTSS therapy [27].

In conclusion, two studies suggest that ambulance workers have a higher risk of mortality and fatal accidents than the general working population. One study indicates a higher relative risk for injury among ambulance workers compared to the national average and the health services in USA, whereas, two studies report high injury rates without comparative data. However, different case definitions make it difficult to compare across the studies.

## Discussion

### Health status in the ambulance services

This analysis demonstrates the wide range of health problems in the ambulance services, such as post-traumatic stress symptoms, mental and somatic problems, injuries, fatal accidents, and infectious diseases.

The prevalence of post-traumatic stress symptoms was high in some studies [1,14,26,29,38]. However, these findings should be interpreted with caution, particularly because the high PTSD symptom scores in the ambulance services might reflect that, when asked to report on a traumatic incident, ambulance workers may have a larger reservoir of potentially traumatic memories to choose from than the general population. Hence, ambulance personnel may score much higher on the PTSD scales than the general population without necessarily having more actual problems. Therefore, more research should focus on sleeping problems, intrusion and hyperarousal among ambulance personnel.

High prevalence rates were also reported for anxiety [1] and general psychopathological problems [14,23,43]. However, because of non-representative samples and inadequate statistical testing it is still unclear whether ambulance personnel experience more mental health problems overall than the general working population.

Studies indicate that ambulance personnel have more somatic health problems, i.e higher blood pressure [18] and more musculoskeletal- and physical health problems than the general population [36,55]. However, although ambulance workers had elevated physiological arousal while running calls, no study has documented a possible "spill over" effect from the stressful period to the remainder of the workday or to after work. One might also question whether neuroendocrine and blood pressure activities are definitive measures of the impact of work exposure on health [65].

Ambulance workers have increased standardized mortality rate [8] and occupational fatality rate [31] and a high ratio of early retirement [40]. This might suggest the existence of a healthy worker effect [66] in the ambulance services. Hence, the actual health problems in the ambulance services might be underestimated. We view this conclusion as tentative because the data are based on only one study for each major outcome variable.

### Work-related and individual correlates

Different mechanisms have been suggested to explain the differences in PTSD symptoms between ambulance workers within the ambulance services. Both degree of work-related stress [17,45,54], and frequency of incident stressors [17], are reported to be significantly correlated with more PTSD symptoms. This in accordance with the findings in a previous review, based on 2 studies, that ambulance workers are at risk of increased PTSD symptoms with greater exposure to critical incidents[7]. However, others have suggested that personality disposition or vulnerability might explain the stability of PTSD symptom caseness across studies [16,23,29,53], but research is scarce and the evidence not convincing.

High psychological stress and low social support are consistently associated with more symptoms of mental health problems, but some studies claim that individual factors [2,19,48] were more highly correlated with psychological strain than are work-related factors. In accordance with this, the previous review reported significant relations between job stress (2 studies), low support (2 studies), in addition to threats to personal safety and health (2 studies), and general mental health problems, based on four studies in all.

In conclusion, there is no clear answer as to what degree work-related factors or individual factors, such as personality factors or individual coping, can explain the health data in ambulance workers, and the specific personality or coping dimensions that reliably predict adverse stress reactions among ambulance workers are still uncertain.

### Methodological shortcomings

Although some studies have used prospective designs or have been based on register data, no clear conclusion can be made about the health status in the ambulance services, and the important work-related and individual correlates. Several methodological challenges, such as small sample sizes, low response rates and non-representative samples limit the value of many studies [7]. Some studies included both paramedics and firefighters, or loosely defined their sample as rescue workers. Moreover, few studies specified the type of ambulance work performed. A model of "front-line" work and advanced life saving may be assumed, but much ambulance work consists of transporting chronically or terminally ill patients between home and hospital.

The specific regional circumstances may be important to the type of ambulance work performed. For example, working in a war zone or in an impoverished urban area may represent quite different challenges and stressors from working in a small city in an industrialized country. Ambulance workers may face different challenges in urban and rural areas, such as differences in the close interaction with the client population, distance to the nearest hospital, number and types of incidents, and overall activity level.

Another methodological challenge concerns the selection and use of appropriate comparison groups. No study has systematically compared the level of symptoms and prevalence of cases between ambulance workers and the normative sample of a relevant working population. Previous research comparing the health status in ambulance personnel with that of general populations has not considered the healthy worker effect [66].

There has also been a lack of consistency in the assessment methods and instruments to measure health problems. Methodologically, the use of different health measures could be seen as an advantage because it gives several indicators of the level of strain in the ambulance services, but it also makes it difficult to compare risk and prevalence with other occupational groups and to draw clear conclusions about the health status of ambulance services across studies. The same problem applies when evaluating the effects of personality and coping behaviour or expectations. Although several studies have argued for an interactionist perspective, there seems to be little consensus on the relevant personality dimensions that confound or moderate, and coping dimensions that moderate or mediate, the relation between work conditions and health outcomes, and how best to address this issue when building models and analysing data. Finally, most studies are retrospective, cross-sectional, or rely on self-report, and it is difficult to empirically support the theoretically expected causal relations or to understand the consequences of acute and chronic stressors over time.

### Strengths, limitations and possible biases

This is the first systematic study to summarize the literature on all aspects of occupational health and health status in the ambulance services. We searched the literature using a number of databases to minimize the risk of overlooking important studies [67]. Nevertheless, it is possible that we did not find all the relevant original studies [68]. The search strategy failed to identify a few studies included in a previous review, because no word for "ambulance workers" was identified in the databases citing these articles. It is also possible that we missed other studies comparing health status in different occupational groups where ambulance workers represent only one of many groups (e.g., data in only one row or column in the tables) [69], as in two of the studies known by the research group [8,9]. However, we believe it unlikely that this bias would change the overall conclusion. Importantly, the study population in this review represents a fairly narrow spectrum of socioeconomic and cultural environments, mainly from Anglo-Saxon and European countries, and it may not be possible to generalize the results to ambulance personnel in other parts of the world.

### Further research

More systematic research is needed to compare the stressor and strain levels experienced by ambulance personnel with those experienced by other similar occupational groups and general working populations. Reporting average scores, caseness, and confidence intervals for ambulance workers and normative working populations would make comparisons between studies more straightforward.

More consistency in the choice of instruments would also help the field. The Impact of Event Scale [70] and other PTSD scales [71] are used most frequently to assess health in studies on ambulance personnel, which allows some comparison between studies. However, one possible concern is that these scales were constructed to screen patients needing treatment in the aftermath of a specific crisis situation and may not be well suited for detecting work stress in general. Maslach's Burnout Inventory [72], an instrument developed for use in health professions, is currently used to study ambulance workers and may represent a starting point for comparing the health status in different ambulance services and health professions.

There is also a lack of alternative health measures to identify smaller, everyday problems in ambulance workers. We suggest some other relevant dimensions as markers of health problems in ambulance workers. Both sleep, which correlates with high work demands and inability to stop thinking about work [73], and the need for recovery [10] are stronger predictors of mental health than work load; problems with either are suggested intermediate steps towards more severe mental health problems.

Surprisingly few studies have investigated physical health, especially musculoskeletal complaints, of ambulance workers, given its high prevalence in population studies [74,75] and considering that ambulance work involves heavy lifting and carrying under difficult conditions. In addition, important health indicators such as alcohol use, suicidal behaviour, help-seeking and sick leave should be addressed.

To further investigate the relation between work environment and health problems among ambulance personnel, both operational and organizational dimensions relevant to ambulance workers should be integrated more systematically. Research should identify how clinical factors of personal importance and the organizational hassles add to the stress of being at the "front line". To address this issue, more attention should be directed to general job pressure (e.g., time pressure, work demands, emotional demands, overtime, and work schedules).

Another important, partly unanswered, question is why, when looking at health differences in a single occupation, some individuals fare worse than others. It is not clear whether these differences relate to personality traits, individual coping skills, or expectations, or whether they are primarily a question of available social support from work colleagues, of good and supportive leadership, or a supportive network outside of work. Furthermore, the effect of potentially important sociodemographic variables is not well understood, for example, how individual variables such as age, sex, and education level affect the level of stress and health problems experienced by ambulance workers.

More theoretically oriented research is needed that includes more epidemiological data to understand the independent contributions of the exposure variables beyond the contribution of the established biomedical risk factors, and the possible interplay of the exposure variables and the biomedical variables. In addition, the psychologists' perspective emphasizing the individuals perception and evaluation of potential harm posed by objective environmental experiences [76] needs to be better integrated in the theoretical and statistical model building. Ambulance work should be studied within the framework of the psychosocial work environment perspective, which includes a focus on ambulance workers' degree of control and influence on the workday and the quality and quantity of informal social interaction in their work. Finally, more prospective studies are needed, most appropriately with baseline measures from just starting ambulance workers, to understand how these different factors relate to ambulance workers' health over time.

## Competing interests

The author(s) declare that they have no competing interests.

## Authors' contributions

TS, ØE and EH jointly conceived the idea for the review. TS performed the literature searches and extracted the data. All authors interpreted the data. TS drafted the manuscript. TS will act as guarantor for the paper. All authors approved the final manuscript.

## Pre-publication history

The pre-publication history for this paper can be accessed here:



## Supplementary Material

Additional File 1 **Health status, work conditions, and individual differences in the ambulance services**. The data (authors; year of publication; site; aims of the study; study design; key explanatory variables, outcome variables; statistical methods; and main results) extracted from the articles. Click here for file
