# Supplementary material for: Health status in the ambulance services: a systematic review
Source: BMC Health Serv Res. 2006 Jul 3;6:82. doi: 10.1186/1472-6963-6-82 (PMC1559607; doi:10.1186/1472-6963-6-82)
Supplement: Additional File 1 — Health status, work conditions, and individual differences in the ambulance services. The data (authors; year of publication; site; aims of the study; study design; key explanatory variables, outcome variables; statistical methods; and main results) extracted from the articles. [file 1472-6963-6-82-S1.doc]

| **Table 1: Health status, work conditions, and individual differences in the ambulance services.** | | | | | |  |  |
| --- | --- | --- | --- | --- | --- | --- | --- |
| Author | Aim | Type of study / | Key explanatory | Outcome | Statistical | Results | Comments |
| Year, |  | data / population / | variables | Measures | methods |  |  |
| Country |  | object number |  |  |  |  |  |
|  |  |  |  |  |  |  |  |
| Aasa U. | Work related psycho- | Cross sectional | Demand, control | Sleeping problems, | Mean, | 25% reported two or more health problems. F sig | Education, years of |
| 2005 | social factors and | self report | social support | headache, and | prevalence | more headache problems than M (48% v. 32%). | employment, |
| Sweden | health complaints | Ambulance personnel | (Karasek model) | stomach symptoms | t-test | sleeping problems(30%), stomach sympt (25%) | physical activity, |
|  |  | N = 1189 (79%) | worry about job |  | Anova | Worry (OR ranging from 1.60- 2.39), and | Smoking, |
|  |  |  | conditions[worry] |  | Logistic reg. | demands (OR range: 1.40-2.41) was sig | no standardized |
|  |  |  | disease/ injury, threats/ | |  | associated with all health outcomes for | instrument on |
|  |  |  | violence, making |  |  | both men and woman, Social support (OR range: | health |
|  |  |  | mistakes) |  |  | 1.39-1.97) only sig for men. Control n.s |  |
|  |  |  |  |  |  |  |  |
| Alexander and | Association | Cross sectional | Critical incidents | Post traumatic | Spearman's rank | GHQ caseness 32% (>5),. MBI high range: | Controlled for |
| Klein | between | Self reported | last 6 month | stress [IES] | correlation | EE 21%, PA 40%, DP 29%. | age, education |
| 2001 | critical incidents | Ambulance personnel | Hardiness (control, | General psycho- |  | Critical incidents r=0.25 with EE. Low score on the | length of service |
| Scotland | and mental health | N=160 (69%) | commitment, | pathology[GHQ-28] | | hardiness subscales d.e+ on burn-out (EE, DP, |  |
|  |  |  | challenge) | Burnout [MBI] |  | and PA): r ranging from .25 to .45 |  |
|  |  |  |  |  |  |  |  |
| Balarajan R | Mortality among men | Census data 1971 | Health sector | Mortality | Standardized | Higher standardized mortality rate than national | Woman not |
| 1989 | employed in the | and 1981 |  |  | mortality | average 109 (CI:101-117) v. 100, | included, |
| U.K | health sector | Ambulance men |  |  | Ratio | lower than own social class 121(CI: 121-122). | no separate data |
|  |  | (N=714) |  |  |  | Causes: all cancer: 233 cases (127 v. 100) | on ambulance |
|  |  |  |  |  |  | ischemic heart disease 266 cases: (116 v.100)) | workers in 1971 |
|  |  |  |  |  |  |  |  |
| Anonymous | Crash related | National highway traffic |  | Crash related | Percentage | 27 ambulance crash related fatalities among | Uncertain |
| 2003 | injuries among EMS | safety administration |  | fatalities |  | ems workers over a 10-year period | number of |
| USA | workers | [NHTSA] fatality analysis | |  |  |  | Ems workers |
|  |  | report system[FARS] |  |  |  |  |  |
|  |  | 1991-2000 |  |  |  |  |  |
| Beaton R. | Social support | Cross- sectional | Job stress, support, | Symptoms of | Path analysis | Job stress d.e+ on job dissatisfaction and strain. | Controlled for |
| 1997 | network conflict and | Self reported | home/work | stress invent- | Correlation | Support at work d.e- on job dissatisfaction | social desirability |
| Washington | work stress / | Firefighters, paramedics | Edwards social | tory. |  | and stress | non representative |
| USA | satisfaction | N = 2050 (50%) | desirability scale | Job dissatisfaction. | | conflict at work d.e+ on stress |  |
|  |  |  |  |  |  |  |  |
| Beaton R. | Effects of traumatic | Prospective, 6 months | Coping response | Post traumatic | factor analysis | IES at t1(R2=.25) and cognitive avoidance | Control for years |
| 1999 | incidents on PTSD | Self- report, register | of rescue workers | stress at T2 [IES] | linear regression | coping(R2=.07) d.e+ on IES at T2 | in service (n.s) |
| USA |  | N =220(45%),T2:N=148 | inventory |  |  | Freq. of events during last year n.s | non representative |
|  |  | firefighters /paramedics | traumatic exp. |  |  |  |  |
|  |  |  |  |  |  |  |  |
|  |  |  |  |  |  |  |  |
| Bennet P et al | Levels of mental | Cross sectional |  | Anxiety, depression | Percentage | 2/3 reported intrusive memories | Controlled for |
| 2004 | health problems | Self reported |  | [HADS], post traum- | | PTSD caseness: 22 (CI 19-26) | gender, education |
| Wales |  | Ambulance personnel |  | atic diagnostic |  | 10% probable clinical level of depression (>11) | cut-off point not |
|  |  | N = 617 (60%) |  | scale [PDS] |  | 22% probable clinical level of anxiety (>11) | given for PDS |
|  |  |  |  | intrusive memories |  |  |  |
|  |  |  |  |  |  |  |  |
| Bennet P et al | Emotional distress | Cross sectional | troubling memories | Anxiety, depression | Percentage | F-inc, D-org, dissociation, and length of service | Controlled for |
| 2005 | in ambulance service | Self reported | Degree and | [HADS], post traum- | | d.e+ on PDS score (R2= .48). (only D-org was | gender, education, |
| Wales |  | Ambulance personnel | frequency of | atic diagnostic |  | associated with caseness OR=1.095). | length of service |
|  |  | N = 617 (60%) | incident stressors | scale [PDS] |  | Home/work, unpredictable work, tension with |  |
|  |  |  | [D-inc / F-inc], and | cognitive appraisal |  | colleagues, and tiredness at work d.e+ on |  |
|  |  |  | org. stressors | questionnaire[CAQ] | | anxiety(R2= .38) and depression(R2= .31) |  |
|  |  |  | [D-org / F-org] |  |  | Incidents involving children were the only |  |
|  |  |  |  |  |  | incident associated with anxiety. |  |
|  |  |  |  |  |  |  |  |
| Boreham C.A | Health status of an | Cross sectional |  | Blood pressure[BP] | Percentage/ | Sig. higher blood pressure than general pop.: | No confounders |
| 1994 | ambulance service | Medical test, Self report |  | Body mass index | Mean | systolic BP (130±1,7 v. 124,8±0.7) |  |
| N-Ireland |  | Ambulance staff |  | coronary risk, | Comparison with | diastolic (87±1.3 v. 78,9±0,5) |  |
|  |  | N = 105 (46%) |  | factors | general | 21% at level of risk(>140mmHg) |  |
|  |  |  |  |  | population | serum cholesterol sig. Lower than in general pop. | |
|  |  |  |  |  |  | but 52% over desirable threshold of 5.2 mmol/l. |  |
|  |  |  |  |  |  |  |  |
| Boudreaux E. | Effects of modifying | Prospective, 2 months | Shift change | Job satisfaction, | Paired t test | Lower EE score at T2, but n.s at T3, and n.s | Control for age, race |
| 1998 | shifts from | and 1year follow up | from 24h to 12h | burnout [MBI] |  | effect on PA or DP at T2 or T3 | gender, education |
| USA | 24 to 12 h | Self reported |  | Schedule attitudes |  | No sig. effect on job satisfaction | income, education |
|  |  | paramedics |  |  |  |  | marital status |
|  |  | N=51 (73%), T2 (50%) |  |  |  |  | experience |
|  |  |  |  |  |  |  |  |
| Boudreaux E. | Job stressors | Cross sectional | Stress diagnostic | Depression, | Correlation | Job stress and depression: r=.34, and | Control for age, race, |
| 1997 | associated with | Self report | inventory | anxiety, and |  | anxiety: r=..38, and global distress: r=.38 | gender, income |
| USA | job satisfaction | Emts | Job satisfaction | general distress |  |  | experience, education |
|  | and health | Study 1:N=61 (87,1%), | (JIG) | [Symptom chkl-90] |  |  | marital status |
|  |  |  |  |  |  |  |  |
|  | Coping | Prospective 1 month | Ways of coping | MBI, Daily stress | Correlation | Blaming oneself / taking to much responsibility, | Not shown how the |
|  | predicting | Self report |  | inventory, Daily |  | confrontive coping (aggression, hostility), and | relative importance |
|  | perceived stress | Emts |  | autonomic nervous | | escape avoidance coping d.e+ on MBI | of coping v. freq of |
|  | and health | T1: N=64 (93%) |  | system inventory |  | and DANSRI, r ranging from .30 to .50 | events is tested. |
|  |  | T2: N=40 (62,5%) |  | (DANSRI) |  |  |  |
|  |  |  |  |  |  |  |  |
|  |  |  |  |  |  |  |  |
|  |  |  |  |  |  |  |  |
|  |  |  |  |  |  |  |  |
| Brough P | Compare trauma | Cross sectional | Organizational-/ | Post traumatic | Structural | Operational hassles d.e+ on IES. | No confounders, |
| 2004 | and organizational | Self reported | operational- | stress [IES] | equation | Organizational hassles d.e- on job satisfaction, | used 8 items on |
| New Zealand | stress in three | Ambulance, police, fire | stressors | Job satisfaction | modelling | and i.e+ on psych strain via low jss | GHQ-12 |
|  | emergency occu- | N= 687(46%) |  | General psycho- |  |  |  |
|  | pations | (232, 223, 231) |  | pathology[GHQ-12] | |  |  |
|  |  |  |  |  |  |  |  |
| Clohessy S. | PTSD symptoms, | Cross sectional | Intrusive memories | Post traumatic | Correlation | PTSD caseness: 21% (DSM-III-R criteria). | No confounders |
| 1999 | intrusive memories | Self report | COPE | Stress symptom | Linear regress. | Depression caseness: 22% | Job stress not |
| U.K | and coping | Paramedics/ technicians | Acute stress | scale [PSS] |  | Response to intrusion: rumination, negative inter- | included. |
|  |  | N = 56 (57%) | Chronic stress |  |  | pretation, suppression, dissociation correlated | Non representative |
|  |  |  |  |  |  | with PTSD and GHQ, r ranging from .39 to .52 |  |
|  |  |  |  |  |  | Wishful thinking r=.45 / .54 with PTSD / GHQ, |  |
|  |  |  |  |  |  | and mental disengagement r= .35 with GHQ |  |
|  |  |  |  |  |  |  |  |
| Chng C.L | Burnout level and | Cross sectional | Sensation-seeking | Burnout (Revicki- |  | 29% sought counselling for job related event | Gender, age |
| 1999 | sensation-seeking | Self report | (AISS) | WRSI) |  | Reported moderate level of BO, mean: 37.7±7.8 | No data given for |
| USA |  | Emts |  |  |  | Reported moderate high AISS, mean 51.8±8.1 | comparison group |
|  |  | N=425(?%) |  |  |  | BO-AISS r=0.18 | non representative |
|  |  |  |  |  |  |  |  |
| Crill M.T. | Back strength and | Cross sectional |  | Back pain | Percentage | 47.8% reported back pain within the last 6 | Convenience sample |
| 2005 | flexibility in EMS | Self reported |  |  |  | months. 39.1% related it to ems work. | no standardised |
| USA | providers | EMS workers |  |  |  | 52.2% reported it to interfere with daily | instrument measuring |
|  |  | N = 90 |  |  |  | activities. | health |
|  |  |  |  |  |  |  |  |
| Cydulka et al | Evaluate stress | Cross sectional | Demographic info | Somatic distress, | variance | High levels of stress (69.3± 6 v. 50 as norm) | No comparison |
| 1997 | levels in ems | Self report | on person and | negative patient | analysis | Somatic distress, mean: 19.6 ±3.3 (>12 high) | with other occu- |
| USA | across USA | Emts | stations. | attitude, job | t-test | Organizational stress, mean:17.3 ± 2.4, | pations, |
|  |  | N=658(22%) |  | dissatisfaction, |  | Negative patient attitude, 17.0 ± 2.6. | only a given value |
|  |  |  |  | organizational- |  | Sig. higher stress in smaller org. | defined as high |
|  |  |  |  | stress |  | and with shorter service, less education and | score |
|  |  |  |  |  |  |  |  |
| Duchateau FX | Exposure to violence | Cross sectional |  | Assaults during | Means, and | One or more assaults were recounted by 23% | no confounders |
| 2002 | during care | Self report |  | career, type of | Percentage | (88% verbal treats, 41% physical treats, |  |
| France |  | EMS providers |  | injury and |  | threatened with gun (13%) and knife (12). |  |
|  |  | N=276(59%) |  | consequences. |  | Type: bruises (40%), wounds (9%), fractures (2%) | |
|  |  |  |  |  |  | Consequences: 4% followed by sick leave, 15% |  |
|  |  |  |  |  |  | by a complaint. 4% reported having got to |  |
|  |  |  |  |  |  | therapy against PTSD |  |
|  |  |  |  |  |  |  |  |
| Gershon, R.M. | Accidents / injuries | Injury / accident reports |  | injury prevalence | prevalence / | Average injury rate of 115 per 100 FT worker | Record data is |
| 1993 | among emergency | Ems workers |  | type of injury, | proportions | overall. Type of injury: Musculoskeletal injuries | based on self-report |
| USA | medical workers | N= 226 injury / accident |  | injury severity, and | | (43%), blood and fluid exposures, and exposure | non repr, |
|  |  | reports (from 197 |  | causes |  | to chemical vapours and fumes (26%) | do not control for |
|  |  | ems workers) |  |  |  | Severity: 24% resulted in 4 or more days lost, | age or gender. |
|  |  |  |  |  |  | 3 cases with surgery (2 permanent, partial |  |
|  |  |  |  |  |  | disability). Causes: handling of stretchers (60%), | |
|  |  |  |  |  |  | toxic or of offensive vapour(11%), other heavy |  |
|  |  |  |  |  |  | lifting (4%), impediments on walkways (8%) |  |
|  |  |  |  |  |  |  |  |
| Grevin, F | PTSD, Ego defense | Cross sectional | Ego defense | Post traumatic | Cut off, t-test, | High PTSD caseness compared with | Age |
| 1996 | mechanisms, and | Self report | mechanism | Stress [PK-scale] | Multivariate | norm 20% v. 5% in Men and 10% in Woman | student v. |
| USA | empathy among | Paramedics/ students | Lifestyle index |  | covariance | Claimed sig. higher denial, repression and lower | experienced workers |
|  | urban paramedics | 225 (?%) | Emotional empathy |  | (compare with | empathy scores than general population | Answer rate |
|  |  |  |  |  | general pop.) | d.e+ of displacement, regression, projection | not given |
|  |  |  |  |  |  | and d.e- of denial on PTSD (R2=. 58) |  |
|  |  |  |  |  |  | d.e- of repression, projection, and d.e+ of |  |
|  |  |  |  |  |  | regression and denial on empathy. |  |
|  |  |  |  |  |  |  |  |
| Hogya PT | Injury profile of | Retrospective review of | Age, sex, education | International | Percentage, | Injury rate: male 50 per 100 FT worker |  |
| 1990 | personnel in a busy | duty record and injury | cause and place of | classification of | wilcoxon rank- | female: 86 per 100 FT worker | not representative |
| USA | urban ems system | records jan 1980 to | injury | disease - 8th rev. | sum test | A total of 93(36%) low back injuries was the |  |
|  |  | june 1983 |  |  |  | most common injury. Remaining injuries were much | |
|  |  | N= 254 injuries |  |  |  | less frequent. Lifting of patients or equipment |  |
|  |  |  |  |  |  | was responsible for 58(62.4%) of low back problems | |
|  |  |  |  |  |  | Overall injury rate: mean 0.83 (for emts), and |  |
|  |  |  |  |  |  | 0.55 (paramedics). Approx 96 injuries accounted | |
|  |  |  |  |  |  | for 481 compensation days, with low back pain |  |
|  |  |  |  |  |  | resulting in 375(78%) days off. |  |
|  |  |  |  |  |  |  |  |
| Johnson, S | Work related stress | Cross sectional |  | Physical health | Mean | Physical health: Ambulance 15.1 v 13.8 norm | No confounders, |
| 2005 | across different | Self report |  | Psychological well- |  | (fire brigade: 12.6 / police: 14.1) | Not reported: |
| U.K | occupations | Occupational groups |  | being (ASSET |  | Psychological well-being: ambulance 20.2 v. | standard errors, |
|  |  | N = 52 ambulance |  | Stress |  | Norm: 18.8 (fire brigade 20.6 / police 19) | Confidence intervals |
|  |  | workers |  | Questionnaire) |  |  | or p-values. |
|  |  |  |  |  |  |  |  |
| Jonsson and | Prevalence of PTSD | Cross sectional | Sense of | Post traumatic | Spearman's rank | Of those who reported a traumatic event | Reports sig. corr. |
| Segesten | and effect of SOC | Self report | coherence [SOC] | Stress [IES] | correlation and | 15% were classified "PTSD cases" (>31) IES | with time in service |
| 2003 |  | Medical technicians and | Traumatic |  | t-test | 12 % PTSD (>5). Effect of close relation to victim | age, education, phys |
| Sweden |  | nurses | incidents |  |  | on PTSD: mean 2.68(2.57) v. 1.19(2.04) | and psych work load |
|  |  | N= 362 (72,4%) |  |  |  | SOC correlates with PTSD (r= -.3) and IES (r=-.4) | but does not control |
| Jonsson and | Association daily-, | Cross sectional | Prof self- | Post traumatic | Mann Whitney | Of those who reported a traumatic event (n=223) | Controls for years in |
| Segesten | traumatic stress and | Self report | description form | Stress [IES] | correlation | 21,5% was classified IES caseness (>26) v. 2.6% | service, marital status |
| 2004 | emotional / mental | Medical technicians and |  |  |  | in the general population. PTSD cases were sign | profession, education |
| Sweden | well-being | Nurses, N= 362 (72,4%) |  |  |  | older and had worked longer in the service. | work schedule |
|  |  |  |  |  |  |  |  |
| Lehman M | Free plasma and | nor/ adrenaline in blood | work-load index | catecholamine | Mean, | Mean differences in Noradrenalin / adrenalin | No confounders |
| 1983 | urine catecholamine | and urine |  | concentration | correlation | in low stress v. high stress situation: | |
| Germany | during medical | protocol |  |  |  | Ambulance men: 22. / 2.6 v. 43.0 / 7.4) ng/min |  |
|  | service | n = 9 ambulance men |  |  |  | physicians: 9.2 / 2.2 v. 27.8 / 8.1 |  |
|  |  | n = 4 physicians |  |  |  | Stress- noradrenalin: r= .58 in ambulance men |  |
|  |  |  |  |  |  | v .88 for physicians (p< .001). Stress - adrenalin: |  |
|  |  |  |  |  |  | r=.27 in ambulance men v. .78 in physicians |  |
|  |  |  |  |  |  |  |  |
| Maguire BJ | Quantify injury risk of | Register | Occupation | Occupational | Rate per 100000 | EMS rate 12.7 compared to working pop 5.0 | No unique code |
| 2002 | emergency service | Ems workers |  | fatality. | per year | (police 14.2 and firefighters 16.2) | for ems workers |
| USA | workers | Minimum estimated |  | [EMS rate] |  | Causes: 67 ground transportation– incidents |  |
|  |  | N = 114 injuries for |  |  |  | 19 air ambulance crashes, 13 cardiovascular |  |
|  |  | ambulance workers |  |  |  | Incidents, 10 homicides, and 5 other causes |  |
|  |  |  |  |  |  |  |  |
| Maguire BJ | Occupational | Retrospective register | cause of injury | Rates of injuries | Standard DOL | Total rate per 100 FT workers:34.6(CI:31.5-37.6) | Convenience sample |
| 2005 | injuries among EMS | data (1998 -2002) | type of injury, body | per 100 FT workers | formula for | The relative risk of injury compared to: | (two urban areas), |
| USA | personnel | Ft EMS workers | part injured, age | per year. | 100 full-time- | national average was 7.0 /CI:6.22-7.87) | and no data on |
|  |  | N = 489 injuries that met | gender, job title, |  | equivalent | health services: 5.8 (CI: 5.12-6.49) | representativity. |
|  |  | U.S department of | lost workdays |  | workers per | fire-fighters: 1.5 (CI: 1.35-1.72) |  |
|  |  | labour criteria’s[DOL] |  |  | year. | Table on age and gender injury rate differences. |  |
|  |  |  |  |  |  |  |  |
| Marmar C | Levels and predictors | Longitudinal (T1: 1,9 / | Traumatic exposure | Post traumatic | Repeated | No difference in traumatic stress comparing | Control for |
| 1999 | of distress in ems at | T2 3,5 years after | Peritraumatic | Stress [IES] | manova | "objective" complex- vs. simpler situations. | elapsed time |
| USA | 2 year follow up | accident). retro- / pro- | emotional stress | Depression, | Multiple reg | Reported changes in mean scores from T1 to T2: | site follow up time |
|  |  | spective. Self report | [PEDS] | anxiety, and |  | IES intrusion 5.36±6.45) v. 4.02±5.85 | occupational groups |
|  |  | Paramedic, fire, police | Peritraumatic | general distress |  | IES avoidance 4.16± 6.38 v. 3.46±5.94 |  |
|  |  | N = 322 | dissociative stress | [Symptom chkl-90] |  | SCL-90 GSI 0.35± 0.31 v. 0.39± 0.35 |  |
|  |  | ( 117 non-response | [PDEQ] |  |  | SCL-90 Somatic. 0.31± 0.30 v 0.35± 0.36 |  |
|  |  | from T1) | Global threat |  |  | Social adjustment 2.01± 0.45 v. 1.86± 0.37 |  |
|  |  |  | to self |  |  | Higher exposure, poor social adjustment |  |
|  |  |  | Social adjustment. |  |  | PEDS, PDEQ, external control |  |
|  |  |  |  |  |  | poor social support, and fewer years in service |  |
|  |  |  |  |  |  | predicted more symptoms at T2, with |  |
|  |  |  |  |  |  | R2 ranging from .15 to .22 |  |
|  |  |  |  |  |  | Only PDEQ predicted change in score |  |
|  |  |  |  |  |  | on all IES dimension, (R2 = .03) |  |
| Mechem, C | Injuries from assaults | Retrospective register |  |  | Means, | 44 of 1100 injury reports from 1996-198 was | no rate of assaults |
| 2002 | on paramedics and | Data 1996-1998 |  |  | percentage | Assaults(4%). Most cases involved paramedics | were reported |
| USA | firefighters | Paramedics, firefighters |  |  |  | (79.5%). Most cases occurred during patient care: | not representative |
|  |  | N = 44 assaults |  |  |  | (93.2%). 36 cases sought help at a |  |
|  |  |  |  |  |  | medical facility, 14 assaults resulted in |  |
|  |  |  |  |  |  | time lost from work |  |
|  |  |  |  |  |  |  |  |
| Mock EF | Anxiety level, effects | 3 month prospective | Verbal and | Anxiety, and | Descriptive | Ambulance v. general working population: | No confounders, |
| 1999 | of violence and shift | Self report | physical | depression. | statistics | State anxiety: 32.6±8,0 v 35.7±10.4, p = .004 | convenience sample |
| USA | schedules | Emts/paramedics | aggression | [Spielbergers | t test, | Trait anxiety: 31.7±7.1 v 34.9±9.2, p= .007 |  |
|  |  | N = 63(81%) | 12 / 24 shift | Stait-trait] | Mann Whitney | Effects of shift and aggressive incident n.s |  |
|  |  |  |  |  |  |  |  |
| Murphy SA | Years of service, | Cross- sectional | years of service | Burnout | Mean | Sig higher average burn-out score in paramedics | controlled for age |
| 1994 | job aspiration and | Self reported |  |  | Bivariat, and | v. firefighters: 20±8.7 v. 17.3±9.2 |  |
| USA | burnout | Firefighters, paramedics |  |  | partial corr. | Years of service sig corr with burnout in |  |
|  |  | N =1983 (50%) |  |  |  | firefighters (r= .06), but n.s in paramedics. |  |
|  |  |  |  |  |  |  |  |
| Öhman U. | Injuries resulting in | Cross sectional | occupation | Medical impair- | Rate per 1000 | impairments: paramedics mean: 2.22 v. mean: | Small number of |
| 2002 | medical impairments | register (1991) |  | ment | per year | 0.38 among all medical personnel, | paramedics, |
| Sweden | in medical personnel | medical personnel |  |  |  | highest rate together with nursing aids | sig level not reported |
|  |  | N=209 (5 paramedics) |  |  |  | working in home care, mean: 0.84 |  |
|  |  |  |  |  |  |  |  |
| Okada, N. | Occupational stress | Cross sectional | Working conditions | Physical strain, | Logistic reg. | 2/3 reports lower back problems v. 17.9% in | No cut-off point for |
| 2005] | among Japanese | Self report | Population density | mental strain |  | general population. 1/3 reported problems with | dependent variable. |
| Region, Japan | Emts | paramedics | Nr. of dispatches |  |  | neck and shoulder. d.e+ of age and qualification | control for length |
|  |  | N=1551 (76.9%) |  |  |  | as paramedic(v. Emts) on mental health problems | of service, age, |
|  |  |  |  |  |  | , but working conditions (n.s) | education |
|  |  |  |  |  |  |  |  |
| Pisarski A. | Social support, shift | Cross sectional | Family support | General psycho- | Structural | Social support from co-workers d.e- on | Experience of shift |
| 2002 | control, coping, | Self report | Supervisor | pathology[GHQ-12] | equation | psychological symptoms. Supervisor support | work, employment |
| Australia | work/home conflict | Ambulance workers | Co-worker | physical health | modelling | i.e- on psych. symptoms via higher shift control | status, work- |
|  | and psychological | N = 60 (37%) | Coping, |  |  | and less work-non-work conflict | schedule |
|  | and physical health |  | work / non w |  |  | Supervisor and family support i.e- on |  |
|  |  |  |  |  |  | psych. symptoms via d.e- on emotion |  |
|  |  |  |  |  |  | focused avoidance coping |  |
|  |  |  |  |  |  |  |  |
| Regehr C. | Post mortem | Cross sectional | Internal control | Post traumatic | Correlation | IES mean 10.67, 15.9% severe range (>26 IES) | No control |
| 2003 | inquires, inter- | Self report | Self efficacy | Stress [IES] | Logistic | d.e+ of length of review on IES (R2=.19) | Regression- |
| Canada | personal relations, | Paramedics, fire | Social supp | Depression[Beck ] | regression | BDI: mean 4.53, 0.8% in severe range | Model not shown |
|  | coping and health | N=264(?%) (86, 186) | Postmortem- |  |  | d.e- of internal control and support of union | Convenience sample |
|  |  |  | reviews |  |  | on BDI (R2= .25) |  |
| Regehr C. | PTSD and disability | Cross sectional | PTSD, BDI | Mental sick leave | t test | 25% taken leave after critical event. | No confounders |
| 2002 |  | Self report | Social support |  | Stepwise | Sig. lower support from family (2.87 v. 3.65) | Convenience sample |
| Canada |  | Paramedic | Social provisions |  | logistic reg. | and overall social provision(79.9 v. 86.8). |  |
|  |  | N= 86(?%) | scale |  |  | Higher score on egocentricity(3.8 v. 1.4) |  |
|  |  |  | Personality |  |  | In regression: d.e+ of egocentricity and social |  |
|  |  |  | [BORTII] |  |  | insecurity on MHS leave (traumatic stress (n.s) |  |
|  |  |  |  |  |  |  |  |
| Regehr C. | Exposure to human | Cross sectional | Critical event | Post traumatic | Mean, | 25.5% in the IES severe range | No confounders, |
| 2002 | tragedy, empathy and | Self report, interview | Social provisions | Stress [IES] | Percentage, | 2.3% in the BDI severe range | convenience sample, |
| Canada | trauma in ambulance | Paramedics | scale | Depression[Beck ] | correlation | social provision d.e- on BDI, not PTSD | cut-off points not |
|  | paramedics | N=86(?%) and 18(int) | Own developed |  |  | n.s effect of spouse family or friends | given |
|  |  |  | support scale |  |  | support on PTSD or BDI |  |
|  |  |  |  |  |  |  |  |
| Revicki, DA | Role clarity, job stress | Prospective 6-months | Strain (WRSI), | Psychological | Linear reg. | WRSI at T1 d.e+ on PDI at T2, (R2= .13). | Control for |
| 1996 | group support, | Self report | role clarity, | distress (PDI) |  | Change in WRSI d.e+ on PDI at T2, (R2=.35). | age, gender, |
| USA | supervisor behaviour | Emts | group support, |  |  | Social support from work group and supervisor | education |
|  | and mental health | N=T1:85(43%) / T2:65 | supervisor behaviour | |  | d.e- on WRSI, (R2=.44 ), role clarity (n.s). | years in work |
|  |  |  |  |  |  |  |  |
| Rodgers L.M. | Compare morbidity | Register | Occupation, | Early retirement | Standardized | SERR ambulance personnel 636 (CI. 558,714) | Control for |
| 1998 | between | Ambulance workers, | length of service, | ratio on | Early retirement | v. 100 (norm), manual: 164 (CI:149-179), | age, gender |
| N-Ireland | ambulance staff and | nurses, (non) manual | year of retirement | medical ground | [SERR] | nursing: 91(CI:75-107), non-manual 38(CI:25-52) |  |
|  | other occupational | workers. |  | [EROMG] |  | EROMG ambulance workers 55,9/1000 v. |  |
|  | groups | N= 534/ 40 ambulance |  |  |  | (manual 24,8, nursing 5,9, non manual 2,6). |  |
|  |  | personnel |  |  |  | Retire at an earlier age compared with manual |  |
|  |  |  |  |  |  | workers, peak 55-59 y. v. 60-64 y. |  |
|  |  |  |  |  |  |  |  |
| Rodgers L.M. | Causes of retirement | Register | Occupation | Medical | Percentage, | No sig different causes of medically retirement. | Control for |
| 1998 |  | Clinical report |  | diagnosis | chi square | Main causes: musculoskeletal- (M37%/F52%), | age, gender. |
| N-Ireland |  | Ambulance workers, |  | ICD code |  | circulatory-(M:25%/F:16%), and mental |  |
|  |  | nurses, (non) manual. |  | (main diagnosis). |  | disorders (M:14%/F:13%). |  |
|  |  | N= 534 |  |  |  | Compared with manual workers ambulance- |  |
|  |  |  |  |  |  | workers are more likely to retire of circulatory |  |
|  |  |  |  |  |  | and mental problems (especially alcohol use: |  |
|  |  |  |  |  |  | 5.1% v. 1.8% and overall 1.7%) |  |
|  |  |  |  |  |  |  |  |
| Shapiro D. | Ambulatory stress | Recordings of blood | Work context, | Systolic / diastolic | Variance | Ambulance cardiovascular measures differed | No confounders |
| 1993 | psychophysiology | pressure and heart rate. | stressful situations | blood pressure | analysis | between ambulance run and station activities: |  |
| USA |  | Self report | and anger | [SBP / DBP] |  | heightened BP and HR on scene, to hospital |  |
|  |  | Other rated incidents |  | heart rate [HR] |  | , and at hospital. Runs rated higher in stress |  |
|  |  | paramedics |  |  |  | by raters were sig. associated with higher |  |
|  |  | N= 33 |  |  |  | SBP mean: 133.2 v. 129.9 |  |
|  |  |  |  |  |  |  |  |
| Sluiter,J.K. | Severity of patient | prospective 3-months | Severity of | Cortisol excretion | Mean, | Severity of patient and time of day (4 - 10 AM) | no confounders |
| 2003 | status predict stress | cortisol | emergency call, and | | variance | predicted raised level of cortisol excretion | short follow up |
| Netherlands | hormone | male ambulance | time of day |  |  | at delivery of patient(4.4 nmol/l) and 15 min | after incident |
|  |  | paramedics=20 |  |  |  | (4.1 nmol/l), 30 min after delivery (3.4 nmol/l) |  |
|  |  |  |  |  |  |  |  |
| Sluiter,J.K. | Work demands, | Cross sectional | Workload | Need for recovery | Multiple | Need of recovery d.e+ on subjective health | control for |
| 2003 | need for recovery, | Self report |  | subjective health | regression | (R2 = .52), physical demands and lack of | gender, age |
| Netherlands | and correlations | ambulance workers |  | complaints (RAND) | | decision latitude d.e+ in step 1(R2=.24), but |  |
|  | with negative health | N=53(65%) (N=3820) |  |  |  | n.s after control for need for recovery. |  |
|  |  |  |  |  |  |  |  |
| Thompson J. | Psychological impact | Cross sectional | Number of | General Health | Mean | Ambulance v. Police: moderate to severe | No control |
| 1993 | of body recovery | Self report | accidents during | Questionnaire | Percentage | category on GHQ 20% v 3%. Sig mean differ- |  |
| U.K | duty | Ambulance workers / | last year | [GHQ] | correlation | rences: GHQ, total.: 6.75±5.76 v. 1.9±3.7 |  |
|  |  | Police (N = 40 / 28) |  | IES |  | IES, avoidance 12.33±8.19 v. 4.7±6.9) |  |
|  |  |  |  |  |  | IES, intrusion 13.80±8.68 v. 7.6± 7.4 |  |
|  |  |  |  |  |  | IES, total 25.88± 15.54 v. 12.3± 12.3 |  |
|  |  |  |  |  |  |  |  |
| Tortella B.J. | Disabling job injuries | Cross sectional |  | SDIH (serious | nr of injuries | 81 SDIHs for a rate of 1 in 31616 dispatches, and | no overall injury |
| 1994 |  | Survey |  | disabling injuries | per year over | 38% of urban EMS systems report at least one | rate nor comparative |
| USA |  | EMS training officers in |  | requiring hospitali- | the number of | SDIH over a one year period. | data was given |
|  |  | 200 most populated U.S |  | sation | responses per | Injury location: hand(23%), head (21%), foot (17%) | |
|  |  | cities |  |  | year | Eye (15%), abdominal(7%), chest(6%) |  |
|  |  | N=88(44%) |  |  |  |  |  |
|  |  |  |  |  |  |  |  |
| Van der Ploeg | Role of acute and | Prospective 1-year | Acute stress, | Burnout [MBI], | t- test, | Caseness: IES(>26): 12%. Fatigue(>76): 10%. | Representative, |
| 2002 | chronic stress: | Self report | chronic stress, | post traumatic | linear regression | Burnout 8.6% v. 5.25% in general pop. | no confounders, |
| Netherlands | predictors of health | paramedics and drivers | and (QEAW)* | stress [IES], | manova | High scores on: EE:12%, DP:18%, Low PA:16% | burnout criteria not |
|  | symptoms reactivity | T1 N=221(56%) | e.g. emotional-, | fatigue [CIS] | repeated | d.e+ of poor communication(R2= .09) and high | given in article. |
|  | and recovery. | T2: N=123 (31%) | physical dem- |  | measures. | emotional demands(R2= .03) at T1 on, IES avoi- | Reference group |
|  |  |  | ands, lack of |  |  | dance at T2, after control for IES at T1(R=.21) | of 1111 Dutch |
|  |  |  | support, auto- |  |  | d.e+ of IES, tot, (R2=.02), poor communication | workers. |
|  |  |  | mi, poor com- |  |  | (R2= .03), physical strain(R2= .03) on MBI-EE | (IES intrusion n.s) |
|  |  |  | munication. |  |  | at T2, after control for MBI-EE at T1(R2= .44). |  |
|  |  |  |  |  |  | d.e+ of lack of support from supervisor on |  |
|  |  |  |  |  |  | DP(R2=.08) and (low) PA (R2=.08), and |  |
|  |  |  |  |  |  | CIS(R2= .03), d.e+ of lack social support from |  |
|  |  |  |  |  |  | colleagues on (low) PA (R2=.07) |  |
|  |  |  |  |  |  |  |  |
|  |  |  |  |  |  |  |  |
|  |  |  |  |  |  |  |  |
|  |  |  |  |  |  |  |  |
| Wastell C.A | Long term effects | Cross sectional | Defense style | Every stress | Linear reg. | D.e+ of suppression of emotions at immature/ | No job stress |
| 2002 | of suppression. | Self report | questionnaire. | and symptom |  | neurotic level (R2, bivariately = .178 / . 221), | no confounders |
| Australia |  | Ambulance officers | Alexithymia | inventory. |  | and d.e+ of inability to be aware of and articulate |  |
|  |  | N = 437(25%) | index. |  |  | emotions (R2= .147), and d.e+ of length of service | |
|  |  |  |  |  |  | (R2=. 029) on stress symptoms. |  |
|  |  |  |  |  |  |  |  |
| Weiss J. | Effect of individual-, | Cross sectional | Basic life support, | Blood pressure | Correlation, | Not higher burnout score than national average | Voluntarily enrolled. |
| 1996 | and work characteris- | Self report, | advanced ALS, | [BP], and pulse. | comparisons | on EE (19.2±10.1) v. (21.0±11.0) and | Controlled for |
| USA | tics on vital sign | electronic monitor | total runs, | Burnout [MBI], | of means | DP(9.3±5.3) v. (8.7±5.9), but a significant | sex, race, |
|  | changes during shift | log book | shift character |  |  | lower PA (28.1±6.5) v. (34.6±7.1) | level of training, |
|  | work. | Emts |  |  |  | No significant change in blood pressure was | marital status |
|  |  | T1 N =69(?%) |  |  |  | seen across shifts. | shift, smoking. |
|  |  | T2 N=40(58% from T1) |  |  |  |  |  |
|  |  |  |  |  |  |  |  |
| Young K.M | Stress outcomes and | Cross sectional | Occupational | Job satisfaction, | t-test v. norm | Claimed significantly higher symptom score on | Scores for reference- |
| 1997 | predictors among | Self report | stress indicator, | mental health, | stepwise reg. | mental and physical health than norm. | group not reported. |
| UK | ambulance workers | Ambulance and ff | type A personality, | physical health. |  | Correlates: | Controlled for sex, |
|  | and firefighters | n=427(29%) | locus of control |  |  | Pressure from relations with others | age, marital status |
|  | compared to a |  | [LOC], |  |  | most important for mental health (R2=. 12) and | number of children, |
|  | normative sample |  | coping |  |  | for physical health (R2 =.20) | age, education, rank |
|  |  |  |  |  |  |  | role, division, length |
|  |  |  |  |  |  |  | of service, time in |
|  |  |  |  |  |  |  | present post |
|  |  |  |  |  |  |  |  |
| Young K.M | Effects of dispute | Longitudinal | OSI (occupation | Mental health, | Stepwise reg. | Claimed to have significantly more symptoms | The level of |
| 1999 | and reorganization. | 6, 12 and 18 months | stress index), | physical health, |  | on physical health problems, but no more | differences is not |
| U.K |  | Self report | ambulance specific | job satisfaction. |  | mental health problems, than in the normative | given for the |
|  |  | Ambulance workers | stress indicators, |  |  | data. Sig more external locus of control, and | comparisons, |
|  |  | N=70 (?) | type A personality, |  |  | more job pressure than in the normative data. | only level of sig. |
|  |  |  | LOC, and coping |  |  | Sig. increase in psychoneurotic symptoms was |  |
|  |  |  |  |  |  | found at t2: perceived inability to exert |  |
|  |  |  |  |  |  | individual influence(R2=.17), type A behaviour |  |
|  |  |  |  |  |  | ( R2 = .26), home/work relationship(R2 = .32) |  |

|  |  |
| --- | --- |
| **Abbreviations:** |  |
| corr. = correlation | M = male |
| CI = 95% confidence interval | n.s = non significant, |
| d.e+ = significant direct positive effect, | OR = odds ratio |
| (i.e. more negative health symptoms) | PA = personal accomplishment |
| d.e- = significant direct negative effect | PTSD = post traumatic stress disorder |
| (i.e. less negative health symptoms). | r = significantly correlated with |
| DP = depersonalization | R2 = explained variance, |
| EE = emotional exhaustion | ref. = reference |
| F = female | regr. = regression |
| i.e+ = significant indirect positive effect | sig. = significant |
| (i.e. more negative health symptoms) | v. = compared to |
| i.e- = significant indirect negative effect | >X = cut of point above X |
| (i.e. less negative health symptoms). | ± X = plus / minus the standard deviation of the mean |
